# DNA methylation dynamics in male germline development in *Brassica Rapa*

**DOI:** 10.1186/s43897-024-00137-9

**Published:** 2025-03-04

**Authors:** Jun Zhang, Di Wu, Yating Zhang, Xiaoqi Feng, Hongbo Gao

**Affiliations:** 1 Shanghai Collaborative Innovation Center of Agri-Seeds, Joint Center for Single Cell Biology, School of Agriculture and Biology, Shanghai Jiao Tong University, Shanghai, 200240 China; 2 Institute of Science and Technology Austria (ISTA), Klosterneuburg, 3400 Austria

**Keywords:** DNA methylation, Methylome, Differentially methylated regions, RNA-directed DNA methylation, Male reproductive development, *Brassica Rapa*

## Abstract

Dynamic DNA methylation represses transposable elements (TEs) and regulates gene activity, playing a pivotal role in plant development. Although substantial progress has been made in understanding DNA methylation reprogramming during germline development in *Arabidopsis thaliana*, whether similar mechanisms exist in other dicot plants remains unclear. Here, we analyzed DNA methylation levels in meiocytes, microspores, and pollens of *Brassica Rapa* using whole-genome bisulfite sequencing (WGBS). Global DNA methylation analysis revealed similar CHH methylation reprogramming compared to *Arabidopsis*, while distinct patterns were observed in the dynamics of global CG and CHG methylation in *B. rapa*. Differentially methylated region (DMR) analysis identified specifically methylated loci in the male sex cells of *B. Rapa* with a stronger tendency to target genes, similar to observations in *Arabidopsis.* Additionally, we found that the activity and genomic targeting preference of the small RNA-directed DNA methylation (RdDM) were altered during *B. Rapa* male germline development. A subset of long terminal repeat (LTR) TEs were activated, possibly due to the dynamic regulation of DNA methylation during male sexual development in *B. Rapa.* These findings provided new insights into the evolution of epigenetic reprogramming mechanisms in plants.

**Supplementary Information:**

The online version contains supplementary material available at 10.1186/s43897-024-00137-9.

## Core

DNA methylation reprogramming during male sexual development has been extensively studied in model plants; however, whether conserved mechanisms and functions exist in *Brassica Rapa* remained unknown. Through the isolation of male sex cells from *B. Rapa*, we discovered the conserved CHH methylation reprogramming event and identified sexual-lineage-specific methylation loci similar to those in *Arabidopsis*. Additionally, our methylome and transcriptome data revealed altered RdDM activity and a burst of transposable elements (TEs) in *B. Rapa* male sex cells.

## Genes & accession numbers

Information for the genes discussed in this article is available in the Genomic Database for *Brassica Rapa* (NHCCDB, http://tbir.njau.edu.cn/NhCCDbHubs/index.jsp) under the accession numbers provided in Supplementary Table S4.

## Introduction

DNA methylation is a major epigenetic modification that can silence TEs, modulate chromatin conformation and gene expression to maintain genome integrity and transcriptional homeostasis in plants and animals (He et al. 2011; Jones 2012; Law and Jacobsen 2010; Zhang et al. 2018). DNA methylation undergo dynamic regulation throughout development, which can be regulated at the establishment, maintenance and removal processes (Zhang et al. 2018). In mammals, DNA methylation occurs mostly in CG context, and DNA methylation reprogramming was observed during development, which was actively regulated by the DNA methyltransferases (Dnmts) and ten-eleven translocation (TET) family of dioxygenases (He et al. 2011; Law and Jacobsen 2010).

While in plants, cytosines methylation occurs in the contexts of CG, CHG and CHH (where H is A, C, or T) and several plant specific methylation pathways were identified, such as the CHROMOMETHYLASE methyltransferase, which functions on the maintenance of non-CG methylation and the RdDM pathway, that mediates de novo methylation by the guidance of small RNA (Du et al. 2015; He et al. 2011; Law and Jacobsen 2010; Pikaard and Mittelsten Scheid 2014). The canonical RdDM pathway has been extensively characterized in *Arabidopsis* through forward genetics and other studies, and the entire mechanism can be briefly divided into two processes, including small RNA production mediated by RNA polymerase IV (Pol IV), RNA-dependent RNA polymerase 2 (RDR2), and Dicer-like protein 3 (DCL3) (Blevins et al. 2015; Singh et al. 2019; Zhai et al. 2015) and targeted methylation mediated by the Argonaute family of proteins, RNA polymerase V (Pol V), and DRM methyltransferases (Cao and Jacobsen 2002; Wierzbicki et al. 2009). Dynamic regulation of DNA methylation by active methylation and demethylation has also been reported in plants. In *Arabidopsis* reproductive development, active DNA demethylation occurs in the central cell and pollen vegetative cells, which lead to TEs activation and generate siRNAs that reinforce TEs methylation in the germ cells (Calarco et al. 2012; Hsieh et al. 2009; Ibarra et al. 2012; Rodrigues and Zilberman 2015; Slotkin et al. 2009). Moreover, through the isolation of the male sex cell at particular stages, the detail of CG and CHG methylation reprogramming was characterized and RdDM mediated sexual-lineage-specific DNA methylation loci (SLMs) was identified, which can regulate gene expression and splicing, and is required for male meiosis (Walker et al. 2018).

It has been shown that dynamic regulation of DNA methylation can be detected in many plant species during plant development and environment (Zhang et al. 2018; Yao et al. 2022), however, the knowledge of cell-type specific DNA methylation and developmental dynamic methylation is still limited*.* Whether the fine-tuned regulatory mechanisms during reproductive development of model plants are conserved in other plants and the biological significance of such cell type specific methylation pattern need to be further investigated. *B. Rapa*, which diverged from *Arabidopsis* approximately 14.5 million years ago (mya), is one of the most representative vegetable crops of *Brassicaceae*, many of which are important horticultural species (Cheng et al. 2017; Tang and Lyons 2012; Warwick et al. 2006). It has shown by phylogenetic studies that homologous genes for most of the components of the RdDM pathway in *Arabidopsis* can be found in *B. Rapa*, suggesting a common regulatory mechanism between the two species (Cao et al. 2016; Feng et al. 2022; Luo et al. 2023; Zhang et al. 2023b). Previous studies also demonstrated a conserved RdDM mediated CHH hypermethylation found in *B. Rapa* embryo (Chakraborty et al. 2021), and the productions of 24-nt siRNAs at hundreds of siren (small-interfering RNA in endosperm) loci in ovule, which were similar observed in *Arabidopsis* and rice (Burgess et al. 2022; Grover et al. 2020; Rodrigues et al. 2013; Zhou et al. 2022). However, whether conserved DNA methylation dynamics during male gametogenesis and SLM mediated gene regulation mechanism exist in *B. Rapa* and other dicot plants remain unclear.

In this report, we generated single-base resolution DNA methylome data from isolated male meiocytes, microspores, and pollens of *B. Rapa*, allowing us to characterize DNA methylation dynamics during male meiosis and gametogenesis. Furthermore, by integrating transcriptome analyses for each cell type, we explored the relationship between DNA methylation and gene/TE expression, offering deeper insights into the regulatory role of DNA methylation reprogramming in *B. Rapa* male germline development.

## Results

### Global DNA methylation reprogramming during male germline development in *B. Rapa*

To explore the DNA methylation dynamics during the male reproductive development of *B. Rapa*, we performed WGBS in the meiocytes, microspores, pollens and leaves of a *B. Rapa* DH line ‘K2’ of Non-heading Chinese cabbage (*B. Rapa* ssp*. chinensis*). The robustness of cell isolation is supported by RNA-seq data for each cell type, which show that genes specifically expressed in each cell type are enriched in the relevant biological processes (Figure S2). Two independent biological replicates of the DNA methylomes for each cell type were generated and the minimum sequencing depth is 6.9-fold coverage (Table S1).

**Fig. 1:**
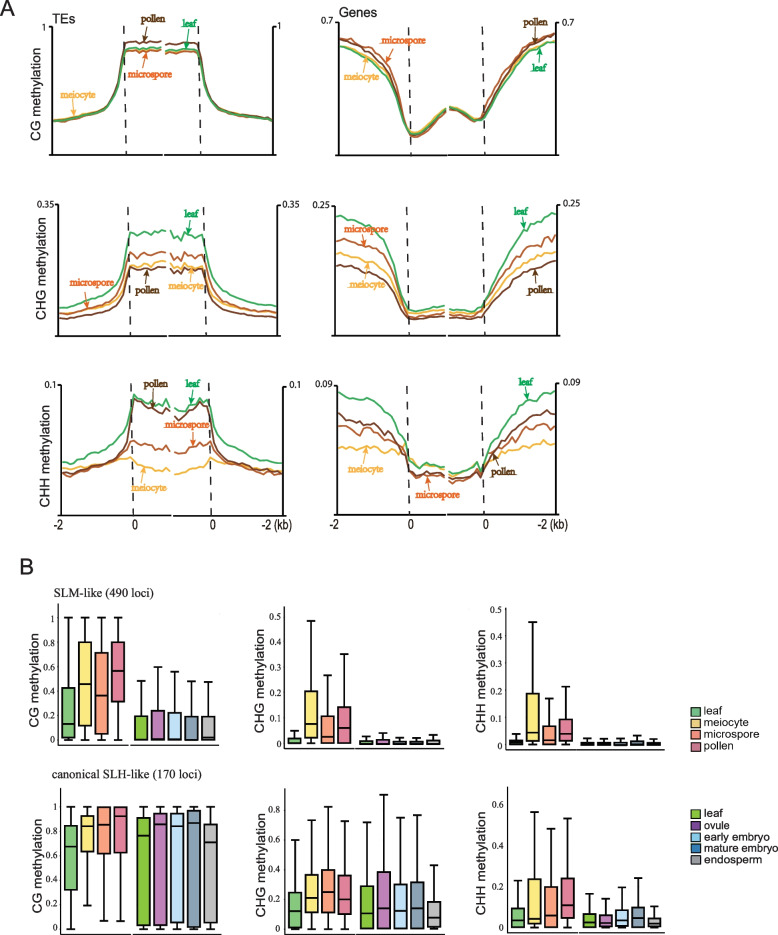
Global DNA methylation of transposons and genes in K2. **A **K2 transposons (left) or genes (right) were aligned at the 5’ and 3’ ends (dashed lines) with average methylation in the CG, CHG or CHH context for each 100-bp interval were plotted for leaf, meiocyte, microspore and pollen. Two biologic replicates were performed for WGBS with leaf or sex cells in K2 (see Methods) CG methylation is shown in the first row, CHG in the middle row, CHH in the last row (**B**) Box plots showing the absolute methylation in K2 leaf, meiocyte, microspore and pollen, and R-o-18 leaf, ovule, early embryo, mature embryo and endosperm at SLM-like loci and canonical SLH-like loci (see text). Two biologic replicates were performed for WGBS with sex cells or leaf tissue in K2 (see Methods), three biologic replicates were analyzed for WGBS with ovule, endosperm or leaf tissue in R-o-18^[25]^, and two biologic replicates were analyzed for WGBS with early or mature embryo in R-o-18^[25]^. Box plots were generated on the basis of 50-bp windows with substantial methylation in all cell types in the corresponding sequence context with the horizontal line marks the median

Global methylation distribution analysis showed that DNA methylation was enriched in TEs and low in gene regions in *B. Rapa* cells, consistent with patterns observed in other plant species. However, unlike the elevated CG methylation seen in *Arabidopsis* male germ cells (Walker et al. 2018), *B. Rapa* meiocytes and microspores exhibited CG methylation levels similar to those in somatic tissues, with an increase observed in mature pollens. This suggests that the reinforcement of TE silencing via CG methylation occurs at later stages of gametogenesis in *B. Rapa*. In addition, CHG methylation in *B. Rapa* initially increased in microspores but decreased in mature pollens, remaining lower than in somatic tissues. This contrasts with *Arabidopsis*, where CHG methylation in male sex cells stays consistently higher than in somatic tissues throughout development (Walker et al. 2018). Notably, a recent study reported a similar pattern of CHG methylation dynamics during gametogenesis in rice (Li et al. 2024), suggesting that different plant species may employ distinct regulatory mechanisms for CHG methylation during male germline development. Despite the differences in CG and CHG methylation dynamics between *Arabidopsis* and *B. Rapa* male germ cells, CHH methylation in *B. Rapa* meiocytes was significantly reduced in meiocyte and gradually re-established during gametogenesis, a pattern similar to that observed in *Arabidopsis* (Walker et al. 2018) and tomato (Lu et al. 2021) (Fig. 1A).

We also investigated global DNA methylation dynamics during other developmental processes by re-analyzing public methylome data from the inbred line R-o-18 (Chakraborty et al. 2021). Analysis of global CG methylation revealed it was elevated in the embryo and reduced in the endosperm, reflecting patterns observed in *Arabidopsis* (Hsieh et al. 2009). However, CHG methylation levels over TEs were significantly lower in the R-o-18 early embryo and endosperm than leaves, whereas in *Arabidopsis*, CHG methylation in these tissues was higher compared to somatic tissues. This indicates a divergence in CHG methylation regulation between the two species. Additionally, a reduction in CHH methylation was observed in R-o-18 ovules compared to other tissues, suggesting that CHH methylation reprogramming may also occur in female sex cells (Figure S3).

These results demonstrate that DNA methylation is dynamically regulated during development in *B. Rapa*. A clear CHH methylation reprogramming event was observed in *B. Rapa* sex cells as early as the meiotic stage, suggesting a conserved CHH methylation remodeling mechanism across plant species. However, the dynamics of CG and CHG methylation during reproductive and embryonic development in *B. Rapa* differed from those in *Arabidopsis*, suggesting that different plant species may employ divergent mechanisms to regulate CG and CHG methylation throughout development.

### Sexual-lineage-specific DNA methylation loci in *B. Rapa*

In *Arabidopsis*, male sex cells possess several hundred SLMs, which regulate gene expression and splicing. These SLMs are generated by tapetum-derived small RNAs, characterized by relaxed targeting stringency (Long et al. 2021). To determine whether SLMs exist in *B. Rapa*, we applied similar comparative analysis on sex cells and somatic tissue (leaves) and identified 660 hypermethylated loci in *B. Rapa* sex cells. Similar to *Arabidopsis*, these hypermethylated loci were relatively small, with an average size of 222 bp. Using the same criteria from previous study in *Arabidopsis* (Walker et al. 2018), where CHH and CHG methylation levels were used as indicator of RdDM activity in somatic tissues, we identified 490 sex-cell-specific hypermethylated loci that show low RdDM activity in somatic tissue (SLM-like loci). Additionally, we found 170 loci that resemble canonical sexual-lineage-hypermethylated loci (SLHs), which are also RdDM targets in somatic tissues but have increased methylation in sex cells. We then plotted the CG, CHG, and CHH methylation levels at both the SLM-like and canonical SLH-like loci in male sex cells, together with methylation levels quantified from public data on leaf, ovule, endosperm, early embryo, and mature embryo of *B. Rapa* R-o-18 at homologous regions (Chakraborty et al. 2021). The SLM-like loci exhibited an increase in methylation levels across all three contexts specifically in male sex cells. In contrast, the methylation levels at the canonical SLH-like loci increased not only in male sex cells but also in ovules and embryos (Fig. 1B). This pattern of tissue-specific methylation dynamics at the SLM/SLH loci mirrors observations in *Arabidopsis*, suggesting that the production of specific DNA methylation loci in the male sexual lineage, as seen in *Arabidopsis*, may be conserved across *Brassicaceae* species.

**Fig. 2:**
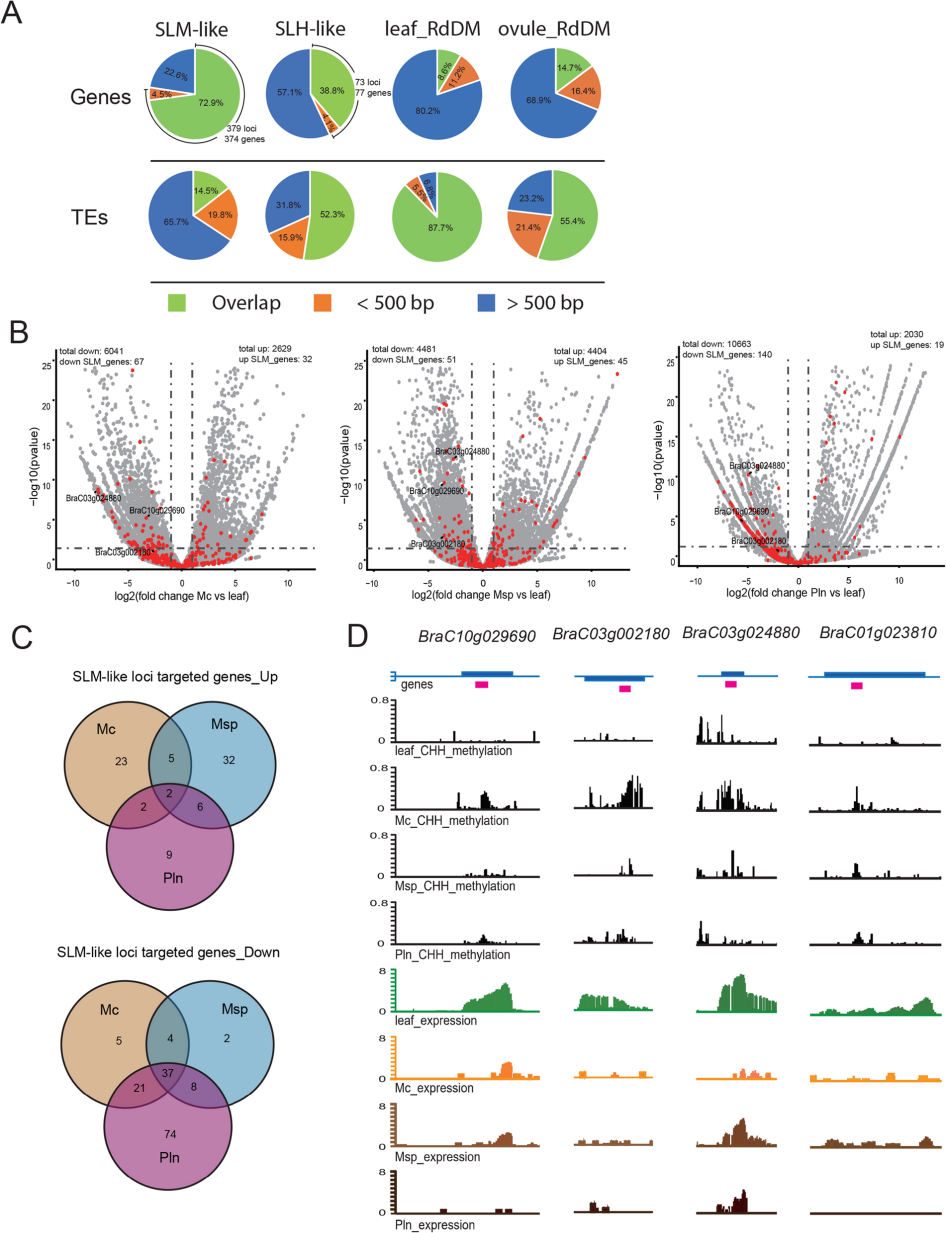
SLM-like loci target and may affect genes expression in male germline. **A **Pie charts displaying percentages of overlapping (green), within 500bp (yellow), and more than 500 bp (blue) from genes and TEs at each category of SLM-like and canonical SLH-like loci and RdDM targeted loci of leaf and ovule in R-o-18. Two biologic replicates were performed for WGBS with sex cells or leaf tissue in K2 (see Methods) Three biologic replicates were analyzed for WGBS with WT or rdr2 ovule, WT or rdr2 leaf in R-o-18^[25]^ . **B **Volcano plot showing the differentially expr-essed genes (DEGs) in meiocyte, microspore and pollen compared with leaf with the SLM-like loci targeted genes highlighted in red. Three biologic replicates were performed for RNA-seq with sex cells or leaf tissue (see Methods). The vertical and horizontal dashed lines show the cutoff of the fold change > 2 and *p*-value < 0.05, respectively; total down/up, the number of total significantly down/up-regulated genes in each group; down/up SLM_genes, the number of significantly down/up-regulated SLM-like loci targeted genes in each group. **C** Veen diagrams showing the overlap of up-regulated and down-regulated genes targeted by SLM-like loci in meiocyte, micr-ospore and pollen. **D** Snapshots showing the CHH methylation ratio and expression level (in log2(TPM+1)) of BraC10g029690, BraC03g002180, BraC03g024880, BraC-01g023810 in K2 leaf, meiocyte, microspore and pollen. Values of the methylomes and transcriptomes are from two and three biologic replicates, respectively. SLM-like loci were highlighted in magenta. Mc, meiocyte; Msp, microspore; Pln, pollen

Unlike RdDM targets in somatic tissues, a typical feature of *Arabidopsis* SLMs is their preference for targeting genes rather than transposons (Walker et al. 2018). Similarly, many SLM-like loci (*n* = 379) and canonical SLH-like loci (*n* = 73) in *B. Rapa* were found to overlap with or be located within 500 bp of genes, in contrast to RdDM target loci in leaf or ovule tissues (Chakraborty et al. 2021) (Fig. 2A). The identified SLM-like loci in *B. Rapa* primarily targeted gene bodies, with the associated genes enriched for the GO terms "binding", "transporter activity" and "catalytic activity" (Figure S5C, Table S2). Therefore, the specific methylated loci in the sex cells of *B. Rapa* may influence cell development by regulating these genes.

To investigate whether the production of SLM-like loci in *B. Rapa* sex cells correlates with target gene expression, RNA-seq analyses were performed on male sex cells isolated using the same method as for the WGBS samples. Three replicates were generated for each cell type, and the consistency between replicates was confirmed (Figure S1B). Differential expression analyses between sex cells and leaves identified that 79 and 151 of the SLM-like loci targeted genes (61.5% in total) were significantly up- or down-regulated in any of the sex cells, respectively (Fig. 2B and C). Notably, SLM-like loci targeting genes that were down-regulated in sex cells showed significant overlap, with 62 (92.5%) of these genes repressed in meiocytes also remaining silenced in later stages. This indicates that the presence of SLM-like loci correlates with stable gene silencing throughout germline development (Fig. 2B and C). In contrast, the up-regulation of SLM-like loci targeted genes was less common in sex cells at different stages, suggesting that the presence of SLM-like loci alone is not sufficient to promote gene transcription. Only 31 (40.2%) of SLH-like loci targeted genes were differentially expressed in sex cells, indicating a minor effect of the SLH loci on gene regulation, as observed in *Arabidopsis* (Figure S5A and B).

Genes that were stably repressed by SLM-like loci exhibited a range of biological functions. Several representative examples include genes involved in biotic stress response (*BraC10g029690*, homologous to Octicosapeptide/Phox/Bem1p family proteins), tip growth regulation (*BraC03g002180*, ortholog of *Arabidopsis* BRISTLED 1), specific metabolic pathways (*BraC03g024880*, homologous to glutathione S-transferase gene family proteins), and cell–cell interaction (*BraC01g023810*, homologous to *Arabidopsis* FERONIA, Fig. 2D). This suggests that SLM/SLH-like loci may contribute to fine-tuning cell type-specific gene expression patterns during gametogenesis in *B. Rapa*, however, more study is needed to verify such transcription regulation.

In summary, we identified sex-lineage-specific DNA methylation loci in *B. Rapa* that resemble those found in *Arabidopsis* in terms of gene-targeting preference and correlation with gene expression, indicating a conserved mechanism underlying the formation of these loci during sexual development. However, there is no functional overlap in the specific genes targeted by these loci in *B. Rapa* and *Arabidopsis*, suggesting these methylation loci may have been adapted to regulate different sets of genes during evolution.

### Altered RdDM activity and unique targeting mechanism during male germline development in *B. Rapa*

**Fig. 3:**
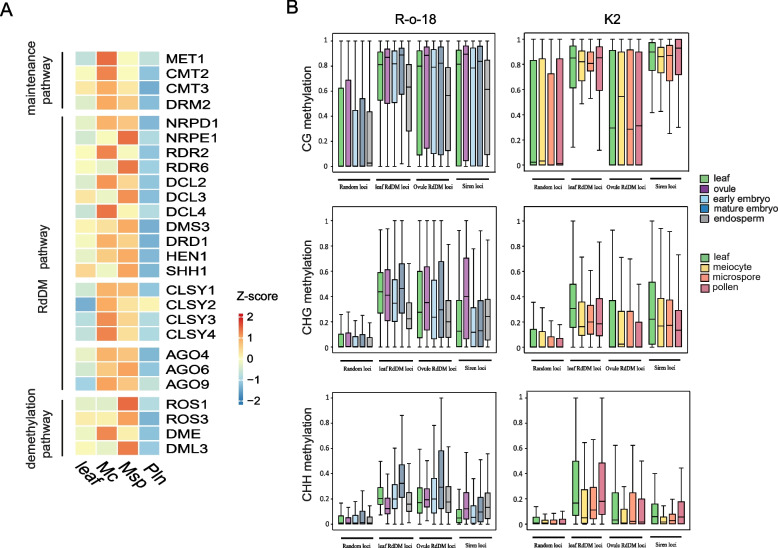
Expression pattern of key genes in DNA methylation and demethylation and DNA methylation at RdDM target loci in K2 male germline. **A **Heatmap showing transcriptome levels of key component genes of the maintenance pathways, RdDM pathway and demethylation pathway in leaf, meiocyte, microspore and pollen of K2. Mc, meiocyte; Msp, microspore; Pln, pollen. Scale bar represents the z-score of the normalized expression values. Three biologic replicates were performed for RNA-seq with sex cells or leaf tissue (see Methods). **B **Box plots illustrating the absolute methylation at 1000 randomly selected loci (mark the ‘Random loci’ on X-axis) in R-o-18^[25]^ (left panel) and their homologous sites in K2 (right panel), leaf RdDM target loci of R-o-18^[25]^ (mark ‘leaf RdDM loci’ on X-axis) (left panel) and their homologous sites in K2 (right panel), ovule RdDM target loci in ovule of R-o-18^[25]^ (mark ‘Ovule RdDM loci’ on X-axis) (left panel) and their homologous sites in K2 (right panel), Siren loci of R-o-18^[25]^ (mark ‘Siren loci’ on X-axis) (left panel) and their homologous sites in K2 (right panel). Two biologic replicates were performed for WGBS with sex cells or leaf tissue in K2 (see Methods), three biologic replicates were analyzed for WGBS with ovule, endosperm or leaf tissue in R-o-18^[25]^, and two biologic replicates were analyzed for WGBS with early or mature embryo in R-o-18^[25]^. Box plots were generated on the basis of 50-bp windows with substantial methylation in all cell types in the corresponding sequence context with the horizontal line marks the median

In *Arabidopsis*, the RdDM pathway plays a crucial role in genome-wide DNA methylation reprogramming and is responsible for the formation of SLMs. To investigate this pathway in *B. Rapa*, we examined the expression of *B. Rapa* orthologs of key components involved in the RdDM pathway. We found that genes in the core machinery, such as *NRPD1*, *NRPE1*, and *DRM2*, showed higher expression in sex cells compared to leaves, suggesting enhanced RdDM activity during male germline development in *B. Rapa*. Additionally, regulatory elements involved in small RNA production and targeting, including *Dicer-like* (*DCL*), *CLASSY*, and *Argonaute* (*AGO*) genes, were significantly activated and exhibited variable expression patterns in male sex cells (Fig. 3A), raising the possibility that RdDM may shift its genomic targeting during this developmental stage. Notably, genes involved in the maintenance of DNA methylation, particularly *MET1* and *CMT2* homologs, as well as those involved in demethylation, including homologs of the *ROS1* family, were also highly active during male germline development in *B. Rapa* (Fig. 3A). This suggests that the dynamic changes in DNA methylation during this process may be influenced by the combined action of de novo methylation, maintenance, and removal of DNA methylation.

To explore whether RdDM targeting mechanisms in male sex cells differ from those in other tissues, we analyzed RdDM-targeted loci in leaf and ovule using public DNA methylation data (Chakraborty et al. 2021). The canonical RdDM target loci identified in leaf exhibited lower methylation levels in male sex cells across all sequence contexts. Additionally, while RdDM-targeted loci in ovules showed high methylation levels in ovules and mature embryos, these loci maintained low methylation levels in male sex cells (Fig. 3B). These results indicate that the canonical RdDM targeting mechanism is altered in the male sex cells of *B. Rapa*.

Female germ cells also exhibit specific DNA methylation through the production of unique siRNA loci, known as siren loci (Grover et al. 2020; Mosher et al. 2009). Of the 191 siren loci identified in R-o-18 genome, 189 were clearly identified in the NHCC001 genome, with only 3 siren loci overlapping with SLM-like or SLH-like loci in male sex cells (Figure S7A), which is consistent with the rare overlap of these loci observed in *Arabidopsis* (Zhou et al. 2022). We also found that CHH methylation, an indicator of siRNA production, was comparably low at the 189 siren loci in male sex cells as in leaves (Figure S7B). Given that siren siRNAs can methylate genomic regions in trans with mismatches (Burgess et al. 2022), we further examined DNA methylation levels at imperfectly matched siren loci (E-value < 1E^−5^, *n* = 1421). In contrast to the enhanced CHH methylation in ovules and endosperm at the siren loci, no elevation in CHH methylation was detected at these loci in male sex cells compared to leaves (Fig. 3B). These data demonstrate that siren loci are largely inactive in male sex cells of *B. Rapa*, supporting previous observations in *Arabidopsis* that small RNA production and RdDM targeting differ between male and female sex cells.

### Dynamic activation of LTR-TEs in *B. Rapa* male sex cells

**Fig. 4:**
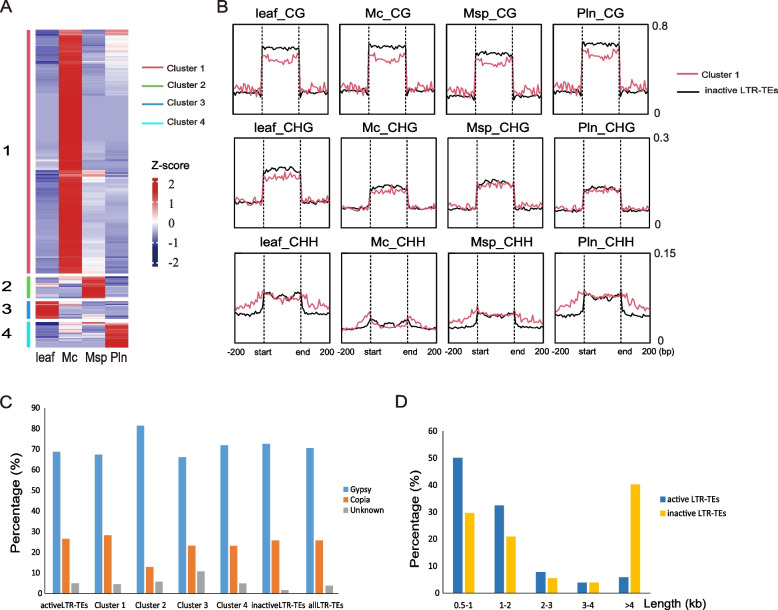
Activation of LTR-TEs in K2 male germline and the surrounding DNA methylation. **A **Heatmap showing the expression level of active LTR-TEs, which can be divided into four clusters, in leaf, meiocyte, microspore and pollen of K2 (left panel). Scale bar represents the z-score of the normalized expression values. Three biologic replicates were performed for RNA-seq with sex cells or leaf tissue (see Methods). **B** Curved line graph displaying the average CG/CHG/CHH methylation ratio around the Cluster1 and inactive LTR-TEs in leaf, meiocyte, microspore and pollen. Two biologic replicates were performed for WGBS with sex cells or leaf tissue in K2 (see Methods). Mc, meiocyte; Msp, microspore; Pln, pollen. **C** The bar charts showing the percentage of each family in active, Cluster 1, Cluster 2, Cluster 3, Cluster 4, inactive and all LTR-TEs. **D** The bar charts showing the percentage of fragment length for active and inactive LTR-TEs

TEs are regulated by multiple mechanisms within the host and can be transiently expressed during development. Previous studies have shown that between ~ 12% and ~ 32.5% of *Arabidopsis* TE genes are expressed in meiocytes, particularly those belonging to LTR-TEs, such as the Gypsy and Copia families (Chen et al. 2010; Yang et al. 2011). Similarly, isolated meiocytes in maize also show a preference for the activation of LTR-TEs (Dukowic-Schulze et al. 2014). To test if TE activation also is the case in *B. Rapa*, we investigated the expression of LTR-TEs in the male sex cell RNA-seq data. Of all 24,764 LTR-TEs analyzed, 992 (4.0%) of them passed the expression threshold of TPM > 10 in at least one cell type and were defined as "active" LTR-TEs. To assess the expression differences between cell types, we performed a k-means clustering analysis and classified these active LTR-TEs into four categories based on expression patterns. The 79.0% active LTR-TEs (Cluster 1, *n* = 784) showed significantly higher activation in meiocyte and 7.1% (Cluster 2, *n* = 70), 5.6% (Cluster 3, *n* = 56) and 8.3% (Cluster 4, *n* = 82) of active LTR-TEs showed highest expression in microspore, leaf and pollen, respectively (Fig. 4A). The majority of active LTR-TEs belong to the Gypsy (*n* = 681) and Copia (*n* = 262) superfamilies. Except for Cluster 2, which exhibited slightly more Gypsy elements activated compared to Copia, the proportion of these two families remained roughly consistent across all clusters (Fig. 4C). 82.5% of the active LTR-TEs were less than 2 kb in length, significantly shorter than inactive LTR-TEs (Fig. 4D). Additionally, the expression levels of active LTR-TEs were much lower than those of protein-coding genes (Figure S6A). These results suggested that these short TEs, or possibly TE fragments, may escape the silencing mechanisms during a specific developmental window.

To investigate the potential link between TE activation and DNA methylation in germ cells, we plotted DNA methylation levels across the Cluster 1 LTR-TEs, which exhibited the highest expression in meiocytes, alongside inactive LTR-TEs for comparison. The results showed that Cluster 1 LTR-TEs exhibited lower CG and CHG methylation across their body regions compared to inactive LTR-TEs. Interestingly, higher CHH methylation was observed in the 200 bp flanking regions of Cluster 1 LTR-TEs in leaves and sex cells, but less prominent in meiocytes. This loss of reinforced flanking CHH methylation in meiocytes was particularly pronounced for Gypsy transposons, which constitute 67.3% of Cluster 1 LTR-TEs (Fig. 4B and Figure S6B). For Cluster 2, 3, and 4 LTR-TEs, the variations in DNA methylation were too large to establish a clear correlation between TE activation and methylation (Figure S6C). Based on these results, it is possible that a subset of LTR-TEs, which were not fully suppressed by robust CG and CHG methylation, were collaboratively silenced by CHH methylation at their flanking regions throughout development. The reprogramming of CHH methylation in male sex cells may disrupt this silencing mechanism, leading to the activation of these TEs. However, further studies are required to elucidate the underling mechanism of LTR-TEs activation in male sex cells, which may involve multiple layers of regulation.

## Discussion

DNA methylation is dynamically regulated throughout plant development and plays a crucial role in transcriptional regulation (Hsieh et al. 2009; Kawakatsu et al. 2016; Park et al. 2016). Although DNA methylation reprogramming during male reproductive development has been extensively studied in *Arabidopsis*, and sexual-lineage-specific DNA methylation has been identified and well-characterized (Calarco et al. 2012; Feng et al. 2013; Ibarra et al. 2012; Walker et al. 2018), the dynamics of DNA methylation during germline development in other dicot plants, as well as the conservation of the mechanisms and functions of sexual-lineage-specific DNA methylation across different plant species, remain to be investigated. Our DNA methylation data revealed low CHH methylation levels in *B. Rapa* meiocytes, which gradually increased at later developmental stages, a pattern similar to that observed in *Arabidopsis*. Furthermore, using comparable approaches, we identified hundreds of sexual-lineage-specific methylation loci in the sex cells of *B. Rapa*, suggesting that conserved mechanisms regulating CHH methylation reprogramming exist within the *Brassicaceae* family. Meanwhile, we found that the dynamics of CG and CHG methylation in *B. Rapa* followed a different trend compared to *Arabidopsis*, suggesting that divergent regulatory mechanisms may be involved. Further studies on DNA methylation dynamics across the *Brassicaceae* family could offer valuable insights into the evolutionary patterns and functional significance of both conserved and divergent mechanisms in DNA methylation regulation.

In *Arabidopsis*, the *CLASSY* family genes regulate the genomic specificity of sRNA production, thereby mediating tissue-specific DNA methylation patterns. Although both male-specific (SLM/SLH loci) and female-specific (siren loci) DNA methylation largely depend on *CLSY3*, the specific methylation loci in male and female sex cells rarely overlap (Long et al. 2021; Zhou et al. 2022, 2018). In conjunction with previously reported data, we demonstrated that SLM/SLH-like loci and siren loci in *B. Rapa* also showed weak correlation. In future studies, our data on male sex cells could be compared with female sex cells and other cell types in *B. Rapa* to further elucidate the diverse regulation of cell-type-specific sRNA production and DNA methylation.

It is conceivable that transposons need to be suppressed to maintain genomic stability in cells that contribute to the next generation (Feng et al. 2013). However, several studies have shown that TEs can become activated during reproductive development, particularly during meiosis (Chen et al. 2010; Dukowic-Schulze et al. 2014; Lu et al. 2021; Yang et al. 2011). In our transcriptome data, we also observed the activation of LTR-TEs and by leveraging DNA methylation data from the same cell types, we found that lower CG and CHG methylation levels over TE bodies, coupled with dynamic CHH methylation at flanking regions, may explain the expression of these TEs. Our transcriptome data showed that *MET1*, *CMT2* and *CMT3* were all activated in meiocyte, however, the effect of the maintenance pathway on shaping the context-specific methylation pattern and TE expression may be limited due to the absence of DNA replication during meiosis. Therefore, RdDM mediated de novo methylation might play a central role in regulating TE expression in meiotic cells. Nevertheless, more in-depth studies are needed to fully elucidate the molecular mechanisms that drive methylation dynamics in different sequence contexts, which may also involve demethylation pathways, and to determine the causal relationship between DNA methylation changes and TE activation.

It needs to be mentioned that detecting DNA methylation profiles in small numbers of cells remains a challenge, as the DNA damage caused by bisulfite conversion can impact genome coverage. Additionally, the enlarged genome of *B. Rapa*, resulting from whole genome duplication and the presence of repetitive sequences, presents greater difficulties for DMR identification compared to *Arabidopsis *(Zhang et al. 2023a; Zou et al. 2023). As such, it is possible that more sexual-lineage-specific methylation loci with subtler DNA methylation variation may exist in the male germline of *B. Rapa*. Therefore, our data could be further explored through the application of innovative bioinformatics strategies and advanced analytical techniques.

## Materials and methods

### Plant materials and growth condition

The seeds of a DH line ‘K2’ of Non-heading Chinese cabbage were sown in Petri dishes that were filled with water-soaked filter paper for germination at 20 °C for 2 days. The germinated seeds of plants were planted in pots containing nutrition soil with matrix and vermiculite (3:1:1 ratio) directly. The plants were grown under 16-h light/8-h dark in a growth chamber at 20 °C, with 75% relative humidity. Four weeks later, four-leaf stage seedlings were moved to another growth room for cold treatment at 4 °C (16-h light/8-h dark, 75% humidity). For meiocytes isolation, stage 10 ~ 11 flower buds were collected and gently squeezed between a slide and a coverslip. The meiocytes (of meiotic prophase I) were released from anthers and then examined carefully under a microscope. Transferring clean meiocytes free from any somatic-cell debris to a new slide with capillary glass pipettes, washing them with 1 × PBS buffer three times and freezing in liquid nitrogen. For isolating microspores at the mononuclear leaning stage, flower buds at 2–2.5 mm were excised and squashed in 1 × PBS. With a stirring bar, the microspores were released from anthers and then filtered through a 40-μm nylon mesh and collected by centrifugation at 200 g for 2 min. Transferring microspores to a new slide with capillary glass pipettes under a stereomicroscope, washing them with 1 × PBS buffer three times and freezing in liquid nitrogen. For isolating pollens, collecting opened flowers and peeling the sepals and petals of the buds. Shaking them in Galbraith buffer (45 mM MgCl_2_, 30 mM sodium citrate, 20 mM MOPS, 1% Triton X-100, adjust pH to 7.0) at 1200 rpm for 3 min and filtering with a 40-μm filter, transferring pollen suspension to a new slide with capillary glass pipettes, washing them with 1 × PBS buffer three times and freezing in liquid nitrogen. For isolating a leaf, using a pair of scissors to cut off a stalk leaf about 1 mm^2^ and freezing in liquid nitrogen.

### Bisulfite sequencing library construction and whole-genome bisulfite sequencing

Cell lysis and bisulfite conversion were performed using the ZYMO EZ DNA Methylation-Gold kit (ZYMO RESEARCH, USA) according to the manufacturer's instructions. Paired-end (150 bp) bisulfite-sequencing libraries for Illumina sequencing were constructed as described earlier (Smallwood et al. 2014). Adaptor-ligated DNA was enriched by 13 cycles of PCR with the following thermal profile: an initial incubation at 95 ◦C for 2 min, 13 cycles at 95 ◦C for 30 s, 65 ◦C for 20 s, 72 ◦C for 30 s and a final incubation at 72 ◦C for 3 min. The enriched libraries were purified with the Ampure XP beads (BECKMAN) prior to quantification with Bioanalyzer (Agilent 5200). Bisulfite libraries were constructed from two biological replicates of WT meiocytes (40 columns per replicate), microspores (about 4.2 k cells per replicate), pollens (about 4 k cells per replicate), leaves (10 ng bisulfite-treated genomic DNA per replicate) of K2 *B. Rapa* in this study. Bisulfite-sequencing data from WT leaf (Chakraborty et al. 2021), ovule(Chakraborty et al. 2021), endosperm (Chakraborty et al. 2021), early embryo (Chakraborty et al. 2021) and mature embryo (Chakraborty et al. 2021) of another *B. Rapa* cultivar (R-o-18) were obtained from published sources. Sequencing was performed at the DNA Sequencing Facility of GENEWIZ biotechnology Co., Ltd. The low-quality sequencing reads, adaptor sequences and the first 9 bp of each read were trimmed using Trim-Galore (Ooi et al. 2017) using default parameters and quality control was performed using FastQC. Trimmed and high-quality reads were aligned to the reference genome of NHCC001 (Li et al. 2020) and R-o-18 (He et al. 2021) using BSmap 1.0 (Xi and Li 2009) (github.com/genome-vendor/bsmap) with the setting: -v 0.15, -w 1, -n 1, -L 100. Genome-wide singleC methylation score of biological replicates were merged after confirming the sample correlation (see Sample Correlation analysis section), and only cytosines with more than 5 informative sequencing reads were retained for further study. For global methylation analysis over genes and TEs, NHCC001- and R-o-18-annotated genes and transposons predicted by the Extensive de-novo TE annotator (EDTA) (Ou et al. 2019) were aligned at the 5' or 3' end. The average methylation was calculated as previously described (Ibarra et al. 2012), using the same settings to avoid skewing the results due to the edges of shorter genes or TEs. LTR-TEs longer than 500 bp were further annotated using DeepTE (Yan et al. 2020), and DNA methylation levels were plotted across different clusters and families of LTR-TEs using EnrichedHeatmap (Gu et al. 2018).

### RNA-seq library construction and transcriptome analysis

Strand-specific RNA-sequencing libraries were prepared according to the protocol described before (Picelli et al. 2014), from three biological replicates of WT meiocytes (20 columns per replicate), microspores (about 2 k cells per replicate), pollens (about 2 k cells per replicate), leaves (about 1.5 mm^2^ tissue per replicate) of K2 *B. Rapa*, respectively.

The libraries were sequenced for paired-end reads of 150 bp using the Illumina platform. Raw reads were trimmed using Trim-Galore (Ooi et al. 2017), followed by alignment to the reference genome of NHCC001 using HISAT2 (Pertea et al. 2016) (daehwankimlab.github.io/hisat2). PCR duplicate fragments were removed using Picard (Tsyganov et al. 2018) (broadinstitute.github.io/picard). Samtools (Danecek et al. 2021) (github.com/samtools/samtools) was used to merge BAM files from biological replicates for subsequent analysis. Correlations between biological replicates were analyzed by the plotCorrelation function based on the output of multiBamSummary (parameter: -bs 500) using Deeptools (Ramírez et al. 2016). Pearson method was used to compute correlation coefficients (Figure S1). Transcript abundance was calculated in transcripts per million (TPM) of mapped reads, with rRNAs filtered out. Raw count processing and differential expression analysis of genes (DEGs) were performed using DESeq2 (Love et al. 2014) with the following thresholds: *p*-value of < 0.05 and |log2FoldChange| of ≥ 1 and Volcano plots were created for visualization using ggplot2 (github.com/tidyverse/ggplot2) package (Klaus and Galensa 2017) Gene expression pattern were demonstrated using Pheatmap (Kolde 2015) (github.com/raivokolde/pheatmap). For TE expression analysis, read counts for each TE were obtained from BAM files using HTSeq (Anders et al. 2015). TEs were defined as active if their TPM value was greater than 10 in at least one tissue, and inactive if less than 0.001 in all tissues. K-means clustering was performed on TPM-normalized data to classify the expression patterns of active TEs, with the number of clusters set to 4, and the results were visualized using Pheatmap.

### Identification of male sex cell specific DNA methylation loci, RdDM target and siren loci

Differentially methylated loci between *B. Rapa* sex cells and leaves were identified according to earlier publication (Walker et al. 2018) with some modifications. Fractional methylation in 50-bp windows across the genome was compared between an average of selected sex cells and leaf tissue. Windows meeting the following criteria: diff_CG > 0 and diff_CHG > 0 and diff_CHH > 0 and (diff_CG + diff_CHG + diff_CHH) > 0.2 were merged to generate larger SLMs if they occurred within 100 bp. Merged SLMs were retained if they covered at least 100 bp, had significantly different levels of total methylation (Fisher’s-exact-test *p*-value of < 0.001) and had higher methylation in all sex cells than in leaves. The 660 sex cell specific hypermethylated loci were further separated into SLM-like (490 loci, CHH and CHG methylation lower than 0.05 and 0.1 in leaves, respectively) and canonical SLH-like loci (170 loci, CHH methylation greater than 0.05 or CHG methylation greater than 0.1 in leaves). Using methylKit (github.com/al2na/methylKit) package to identify the RdDM target loci in leaf and ovule by comparing fractional CHH methylation differences across the genome between the *rdr2* mutant and WT (Chakraborty et al. 2021). Only cytosine covered by at least five reads was considered an effective site. DMRs were searched using a 50-bp sliding window with a 50-bp step size. Windows with at least ten effective cytosines were retained. Finally, regions with q-value of < 0.005 and percent methylation difference larger than 10% for CHH were retained for subsequent analysis. The homologous loci in K2 *B. Rapa* were identified by BLASTN using the following parameters: E-value of ≤ 1E^−5^, identity of ≥ 90. The siren loci were downloaded from earlier report (Grover et al. 2020) and the homologous region in NHCC001 genome (Li et al. 2020) were identified by BLASTN using the following parameters: E-value of ≤ 1E^−5^, identity of ≥ 90.

### Identification of homologous genes and GO analysis

For identification of key genes involved in DNA methylation, protein sequence of each *Brassica* gene from earlier collinear studies (Feng et al. 2022; Zhang et al. 2023b) were downloaded and orthologs in the NHCC001 genome were identified by BLASTP (parameters: E-value of ≤ 1E^−5^). For GO analysis on SLM targeted genes and cell type specific genes, BLASTP (parameters: E-value of ≤ 1E^−5^) were used to search for homologs in the protein database from TAIR (www.Arabidopsis.org). GO analyses of homologous genes were performed by using agriGO v2.0 (systemsbiology.cau.edu.cn/agriGOv2 /index.php) with default parameters and bubble diagrams were generated by ggplot2 (Klaus and Galensa 2017) package in Rstudio.

## Supplementary Information

Additional file 1: Figure S1. The sample correlations for WGBS and RNA-seq analysis. Figure S2. Specifically expressed genes enriched in different cell types. Figure S3. DNA methylation of transposons and genes in R-o-18. Figure S4. The CG/CHG/CHH methylation pattern of SLM-like loci targeted genes in male germline. Figure S5. SLM/SLH-like loci target and may affect genes expression in male germline. Figure S6. The surrounding DNA methylation of active and inactive LTR-TEs in K2 male germline. Figure S7. CHH methylation in the homologous sites of Siren loci in K2.

Additional file 2: Table S1. Summary of MethylC-seq, RNA-seq. Table S2. The number of GO term of SLM-like loci targeted genes. Table S3. The overview of TE annotation by EDTA. Table S4. Data set including gene expression in different male cell types.

## Data Availability

The obtained sequencing data that support the findings of this study have been deposited in the National Center for Biotechnology Information Gene Expression Omnibus (GEO) database under accession number GSE248075.

## Abbreviations

AGO: Argonaute
*B. Rapa*: *Brassica rapa*
DMR: Differentially methylated region
Dnmts: DNA methyltransferases
DCL3: Dicer-like protein 3
LTR: Long terminal repeat
mya: Million years ago
Pol IV: RNA polymerase IV
Pol V: RNA polymerase V
RdDM: RNA-directed DNA methylation
RDR2: RNA-dependent RNA polymerase 2
SLMs: Sexual-lineage-specific DNA methylation loci
SLHs: Sexual-lineage-hypermethylated loci
Siren: Small-interfering RNA in endosperm
TE: Transposable element
TET: Ten-eleven translocation
WGBS: Whole-genome bisulfite sequencing

## References

[CR1] Anders S, Pyl PT, Huber W. HTSeq–a Python framework to work with high-throughput sequencing data. Bioinformatics. 2015;31:166–9. 10.1093/bioinformatics/btu638. 25260700 10.1093/bioinformatics/btu638 PMC4287950

[CR2] Blevins T, Podicheti R, Mishra V, et al. Identification of Pol IV and RDR2-dependent precursors of 24 nt siRNAs guiding de novo DNA methylation in Arabidopsis. Elife. 2015;4:e09591. 10.7554/eLife.09591. 26430765 10.7554/eLife.09591 PMC4716838

[CR3] Burgess D, Chow HT, Grover JW, et al. Ovule siRNAs methylate protein-coding genes in trans. Plant Cell. 2022;34:3647–64. 10.1093/plcell/koac197. 35781738 10.1093/plcell/koac197 PMC9516104

[CR4] Calarco JP, Borges F, Donoghue MT, et al. Reprogramming of DNA methylation in pollen guides epigenetic inheritance via small RNA. Cell. 2012;151:194–205. 10.1016/j.cell.2012.09.001. 23000270 10.1016/j.cell.2012.09.001 PMC3697483

[CR5] Cao X, Jacobsen SE. Role of the arabidopsis DRM methyltransferases in de novo DNA methylation and gene silencing. Curr Biol. 2002;12:1138–44. 10.1016/s0960-9822(02)00925-9. 12121623 10.1016/s0960-9822(02)00925-9

[CR6] Cao JY, Xu YP, Li W, et al. Genome-wide identification of dicer-like, argonaute, and RNA-dependent RNA polymerase gene families in brassica species and functional analyses of their Arabidopsis homologs in resistance to sclerotinia sclerotiorum. Front Plant Sci. 2016;7:1614. 10.3389/fpls.2016.01614. 27833632 10.3389/fpls.2016.01614 PMC5081487

[CR7] Chakraborty T, Kendall T, Grover JW, et al. Embryo CHH hypermethylation is mediated by RdDM and is autonomously directed in Brassica rapa. Genome Biol. 2021;22:140. 10.1186/s13059-021-02358-3. 33957938 10.1186/s13059-021-02358-3 PMC8101221

[CR8] Chen C, Farmer AD, Langley RJ, et al. Meiosis-specific gene discovery in plants: RNA-Seq applied to isolated Arabidopsis male meiocytes. BMC Plant Biol. 2010;10:280. 10.1186/1471-2229-10-280. 21167045 10.1186/1471-2229-10-280 PMC3018465

[CR9] Cheng F, Liang J, Cai C, et al. Genome sequencing supports a multi-vertex model for Brassiceae species. Curr Opin Plant Biol. 2017;36:79–87. 10.1016/j.pbi.2017.01.006. 28242534 10.1016/j.pbi.2017.01.006

[CR10] Danecek P, Bonfield JK, Liddle J, et al. Twelve years of SAMtools and BCFtools. GigaScience. 2021;10. 10.1093/gigascience/giab008. 10.1093/gigascience/giab008 PMC7931819 33590861

[CR11] Du J, Johnson LM, Jacobsen SE, et al. DNA methylation pathways and their crosstalk with histone methylation. Nat Rev Mol Cell Biol. 2015;16:519–32. 10.1038/nrm4043. 26296162 10.1038/nrm4043 PMC4672940

[CR12] Dukowic-Schulze S, Sundararajan A, Mudge J, et al. The transcriptome landscape of early maize meiosis. BMC Plant Biol. 2014;14:118. 10.1186/1471-2229-14-118. 24885405 10.1186/1471-2229-14-118 PMC4032173

[CR13] Feng AN, Kang Z, Ling-Kui Z, et al. Genome-wide identification, evolutionary selection, and genetic variation of DNA methylation-related genes in Brassica rapa and Brassica oleracea. J Integr Agric. 2022;21:1620–32. 10.1016/S2095-3119(21)63827-3.

[CR14] Feng X, Zilberman D, Dickinson H. A conversation across generations: soma-germ cell crosstalk in plants. Dev Cell. 2013;24:215–25. 10.1016/j.devcel.2013.01.014. 23410937 10.1016/j.devcel.2013.01.014

[CR15] Grover JW, Burgess D, Kendall T, et al. Abundant expression of maternal siRNAs is a conserved feature of seed development. Proc Natl Acad Sci. 2020;117:15305–15. 10.1073/pnas.2001332117. 32541052 10.1073/pnas.2001332117 PMC7334491

[CR16] Gu Z, Eils R, Schlesner M, et al. EnrichedHeatmap: an R/Bioconductor package for comprehensive visualization of genomic signal associations. BMC Genomics. 2018;19:234. 10.1186/s12864-018-4625-x. 29618320 10.1186/s12864-018-4625-x PMC5885322

[CR17] He XJ, Chen T, Zhu JK. Regulation and function of DNA methylation in plants and animals. Cell Res. 2011;21:442–65. 10.1038/cr.2011.23. 21321601 10.1038/cr.2011.23 PMC3152208

[CR18] He Z, Ji R, Havlickova L, et al. Genome structural evolution in Brassica crops. Nature Plants. 2021;7:757–65. 10.1038/s41477-021-00928-8. 34045706 10.1038/s41477-021-00928-8

[CR19] Hsieh TF, Ibarra CA, Silva P, et al. Genome-wide demethylation of Arabidopsis endosperm. Science. 2009;324:1451–4. 10.1126/science.1172417. 19520962 10.1126/science.1172417 PMC4044190

[CR20] Ibarra CA, Feng X, Schoft VK, et al. Active DNA demethylation in plant companion cells reinforces transposon methylation in gametes. Science. 2012;337:1360–4. 10.1126/science.1224839. 22984074 10.1126/science.1224839 PMC4034762

[CR21] Jones PA. Functions of DNA methylation: islands, start sites, gene bodies and beyond. Nat Rev Genet. 2012;13:484–92. 10.1038/nrg3230. 22641018 10.1038/nrg3230

[CR22] Kawakatsu T, Stuart T, Valdes M, et al. Unique cell-type-specific patterns of DNA methylation in the root meristem. Nat Plants. 2016;2:16058. 10.1038/nplants.2016.58. 27243651 10.1038/nplants.2016.58 PMC4855458

[CR23] Klaus, and Galensa. ggplot2: elegant graphics for data analysis (2nd ed.). Computing Reviews. 2017.

[CR24] Kolde R. pheatmap: pretty heatmaps. 2015.

[CR25] Law JA, Jacobsen SE. Establishing, maintaining and modifying DNA methylation patterns in plants and animals. Nat Rev Genet. 2010;11:204–20. 10.1038/nrg2719. 20142834 10.1038/nrg2719 PMC3034103

[CR26] Li Y, Liu GF, Ma LM, et al. A chromosome-level reference genome of non-heading Chinese cabbage [Brassica campestris (syn. Brassica rapa) ssp. chinensis]. Hortic Res. 2020;7:212. 10.1038/s41438-020-00449-z. 33372175 10.1038/s41438-020-00449-z PMC7769993

[CR27] Li X, Zhu B, Lu Y, et al. DNA methylation remodeling and the functional implication during male gametogenesis in rice. Genome Biol. 2024;25:84. 10.1186/s13059-024-03222-w. 38566207 10.1186/s13059-024-03222-w PMC10985897

[CR28] Long J, Walker J, She W, et al. Nurse cell derived small RNAs define paternal epigenetic inheritance in Arabidopsis. Science. 2021;373:eabh0556. 10.1126/science.abh0556. 34210850 10.1126/science.abh0556

[CR29] Love MI, Huber W, Anders S. Moderated estimation of fold change and dispersion for RNA-seq data with DESeq2. Genome Biol. 2014;15:550. 10.1186/s13059-014-0550-8. 25516281 10.1186/s13059-014-0550-8 PMC4302049

[CR30] Lu Y, Song Y, Liu L, et al. DNA methylation dynamics of sperm cell lineage development in tomato. Plant J. 2021;105:565–79. 10.1111/tpj.15098. 33249677 10.1111/tpj.15098

[CR31] Luo D, Mei D, Wei W, et al. Identification and phylogenetic analysis of the R2R3-MYB subfamily in Brassica napus. Plants. 2023;12:886. 36840234 10.3390/plants12040886 PMC9962269

[CR32] Mosher RA, Melnyk CW, Kelly KA, et al. Uniparental expression of PolIV-dependent siRNAs in developing endosperm of Arabidopsis. Nature. 2009;460:283–6. 10.1038/nature08084. 19494814 10.1038/nature08084

[CR33] Ooi CC, Mantalas GL, Koh W, et al. High-throughput full-length single-cell mRNA-seq of rare cells. PLoS ONE. 2017;12:e0188510. 10.1371/journal.pone.0188510. 29186152 10.1371/journal.pone.0188510 PMC5706670

[CR34] Ou S, Su W, Liao Y, et al. Benchmarking transposable element annotation methods for creation of a streamlined, comprehensive pipeline. Genome Biol. 2019;20:275. 10.1186/s13059-019-1905-y. 31843001 10.1186/s13059-019-1905-y PMC6913007

[CR35] Park K, Kim MY, Vickers M, et al. DNA demethylation is initiated in the central cells of Arabidopsis and rice. Proc Natl Acad Sci. 2016;113:15138–43. 10.1073/pnas.1619047114. 27956642 10.1073/pnas.1619047114 PMC5206524

[CR36] Pertea M, Kim D, Pertea GM, et al. Transcript-level expression analysis of RNA-seq experiments with HISAT, StringTie and Ballgown. Nat Protoc. 2016;11:1650–67. 10.1038/nprot.2016.095. 27560171 10.1038/nprot.2016.095 PMC5032908

[CR37] Picelli S, Faridani OR, Björklund AK, et al. Full-length RNA-seq from single cells using Smart-seq2. Nat Protoc. 2014;9:171–81. 10.1038/nprot.2014.006. 24385147 10.1038/nprot.2014.006

[CR38] Pikaard CS, Mittelsten SO. Epigenetic regulation in plants. Cold Spring Harb Perspect Biol. 2014;6: a019315. 10.1101/cshperspect.a019315. 25452385 10.1101/cshperspect.a019315 PMC4292151

[CR39] Ramírez F, Ryan DP, Grüning B, et al. deepTools2: a next generation web server for deep-sequencing data analysis. Nucleic Acids Res. 2016;44:W160–5. 10.1093/nar/gkw257. 27079975 10.1093/nar/gkw257 PMC4987876

[CR40] Rodrigues JA, Ruan R, Nishimura T, et al. Imprinted expression of genes and small RNA is associated with localized hypomethylation of the maternal genome in rice endosperm. Proc Natl Acad Sci U S A. 2013;110:7934–9. 10.1073/pnas.1306164110. 23613580 10.1073/pnas.1306164110 PMC3651473

[CR41] Rodrigues JA, Zilberman D. Evolution and function of genomic imprinting in plants. Genes Dev. 2015;29:2517–31. 10.1101/gad.269902.115. 26680300 10.1101/gad.269902.115 PMC4699382

[CR42] Singh J, Mishra V, Wang F, et al. Reaction Mechanisms of Pol IV, RDR2, and DCL3 Drive RNA Channeling in the siRNA-Directed DNA Methylation Pathway. Mol Cell. 2019;75:576–589.e575. 10.1016/j.molcel.2019.07.008. 31398324 10.1016/j.molcel.2019.07.008 PMC6698059

[CR43] Slotkin RK, Vaughn M, Borges F, et al. Epigenetic reprogramming and small RNA silencing of transposable elements in pollen. Cell. 2009;136:461–72. 10.1016/j.cell.2008.12.038. 19203581 10.1016/j.cell.2008.12.038 PMC2661848

[CR44] Smallwood SA, Lee HJ, Angermueller C, et al. Single-cell genome-wide bisulfite sequencing for assessing epigenetic heterogeneity. Nat Methods. 2014;11:817–20. 10.1038/nmeth.3035. 25042786 10.1038/nmeth.3035 PMC4117646

[CR45] Tang H, Lyons E. Unleashing the genome of brassica rapa. Front Plant Sci. 2012;3:172. 10.3389/fpls.2012.00172. 22866056 10.3389/fpls.2012.00172 PMC3408644

[CR46] Tsyganov K, James Perry A, Kenneth Archer S, et al. RNAsik: a pipeline for complete and reproducible RNA-seq analysis that runs anywhere with speed and ease. J Open Source Softw. 2018;3:583. 10.21105/joss.00583.

[CR47] Walker J, Gao H, Zhang J, et al. Sexual-lineage-specific DNA methylation regulates meiosis in Arabidopsis. Nat Genet. 2018;50:130–7. 10.1038/s41588-017-0008-5. 29255257 10.1038/s41588-017-0008-5 PMC7611288

[CR48] Warwick SI, Francis A, Al-Shehbaz IA. Brassicaceae: Species checklist and database on CD-Rom. Plant Syst Evol. 2006;259:249–58. 10.1007/s00606-006-0422-0.

[CR49] Wierzbicki AT, Ream TS, Haag JR, et al. RNA polymerase V transcription guides ARGONAUTE4 to chromatin. Nat Genet. 2009;41:630–4. 10.1038/ng.365. 19377477 10.1038/ng.365 PMC2674513

[CR50] Xi Y, Li W. BSMAP: whole genome bisulfite sequence MAPping program. BMC Bioinformatics. 2009;10:232. 10.1186/1471-2105-10-232. 19635165 10.1186/1471-2105-10-232 PMC2724425

[CR51] Yan H, Bombarely A, Li S. DeepTE: a computational method for de novo classification of transposons with convolutional neural network. Bioinformatics. 2020;36:4269–75. 10.1093/bioinformatics/btaa519. 32415954 10.1093/bioinformatics/btaa519

[CR52] Yang H, Lu P, Wang Y, et al. The transcriptome landscape of Arabidopsis male meiocytes from high-throughput sequencing: the complexity and evolution of the meiotic process. Plant J. 2011;65:503–16. 10.1111/j.1365-313X.2010.04439.x. 21208307 10.1111/j.1365-313X.2010.04439.x

[CR53] Yao Z, Yuan L, Liu K, et al. Warming-induced changes of broccoli head to cauliflower-like curd in Brassica oleracea are regulated by DNA methylation as revealed by methylome and transcriptome co-profiling. Mol Hortic. 2022;2:26. 10.1186/s43897-022-00047-8. 10.1186/s43897-022-00047-8 PMC10515005 37789398

[CR54] Zhai J, Bischof S, Wang H, et al. A one precursor one siRNA model for Pol IV-dependent siRNA biogenesis. Cell. 2015;163:445–55. 10.1016/j.cell.2015.09.032. 26451488 10.1016/j.cell.2015.09.032 PMC5023148

[CR55] Zhang H, Lang Z, Zhu J-K. Dynamics and function of DNA methylation in plants. Nat Rev Mol Cell Biol. 2018;19:489–506. 10.1038/s41580-018-0016-z. 29784956 10.1038/s41580-018-0016-z

[CR56] Zhang J, Ahmad M, and Gao H. Application of single-cell multi-omics approaches in horticulture research. Mol Hortic. 2023a;3:18. 10.1186/s43897-023-00067-y. 10.1186/s43897-023-00067-y PMC10521458 37789394

[CR57] Zhang L, Yang B, Zhang C, et al. Genome-wide identification and posttranscriptional regulation analyses elucidate roles of key argonautes and their miRNA triggers in regulating complex yield traits in rapeseed. Int J Mol Sci. 2023b;24:2543. 10.3390/ijms24032543 PMC9916703 36768865

[CR58] Zhou M, Coruh C, Xu G, et al. The CLASSY family controls tissue-specific DNA methylation patterns in Arabidopsis. Nat Commun. 2022;13:244. 10.1038/s41467-021-27690-x. 35017514 10.1038/s41467-021-27690-x PMC8752594

[CR59] Zhou M, Palanca AMS, Law JA. Locus-specific control of the de novo DNA methylation pathway in Arabidopsis by the CLASSY family. Nat Genet. 2018;50:865–73. 10.1038/s41588-018-0115-y. 29736015 10.1038/s41588-018-0115-y PMC6317521

[CR60] Zou Y, Wang J, Peng D, et al. Multi-integrated genomic data for Passiflora foetida provides insights into genome size evolution and floral development in Passiflora. Mol Hortic. 2023;3:27. 10.1186/s43897-023-00076-x. 10.1186/s43897-023-00076-x PMC10726625 38105261

