# Supplementary material for: DNA methylation dynamics in male germline development in Brassica Rapa
Source: Mol Hortic. 2025 Mar 4;5:16. doi: 10.1186/s43897-024-00137-9 (PMC11877836; doi:10.1186/s43897-024-00137-9)
Supplement: Supplementary file 1 — Additional file 1: Figure S1. The sample correlations for WGBS and RNA-seq analysis. Figure S2. Specifically expressed genes enriched in different cell types. Figure S3. DNA methylation of transposons and genes in R-o-18. Figure S4. The CG/CHG/CHH methylation pattern of SLM-like loci targeted genes in male germline. Figure S5. SLM/SLH-like loci target and may affect genes expression in male germline. Figure S6. The surrounding DNA methylation of active and inactive LTR-TEs in K2 male germline. Figure S7. CHH methylation in the homologous sites of Siren loci in K2. [file 43897_2024_137_MOESM1_ESM.pdf]

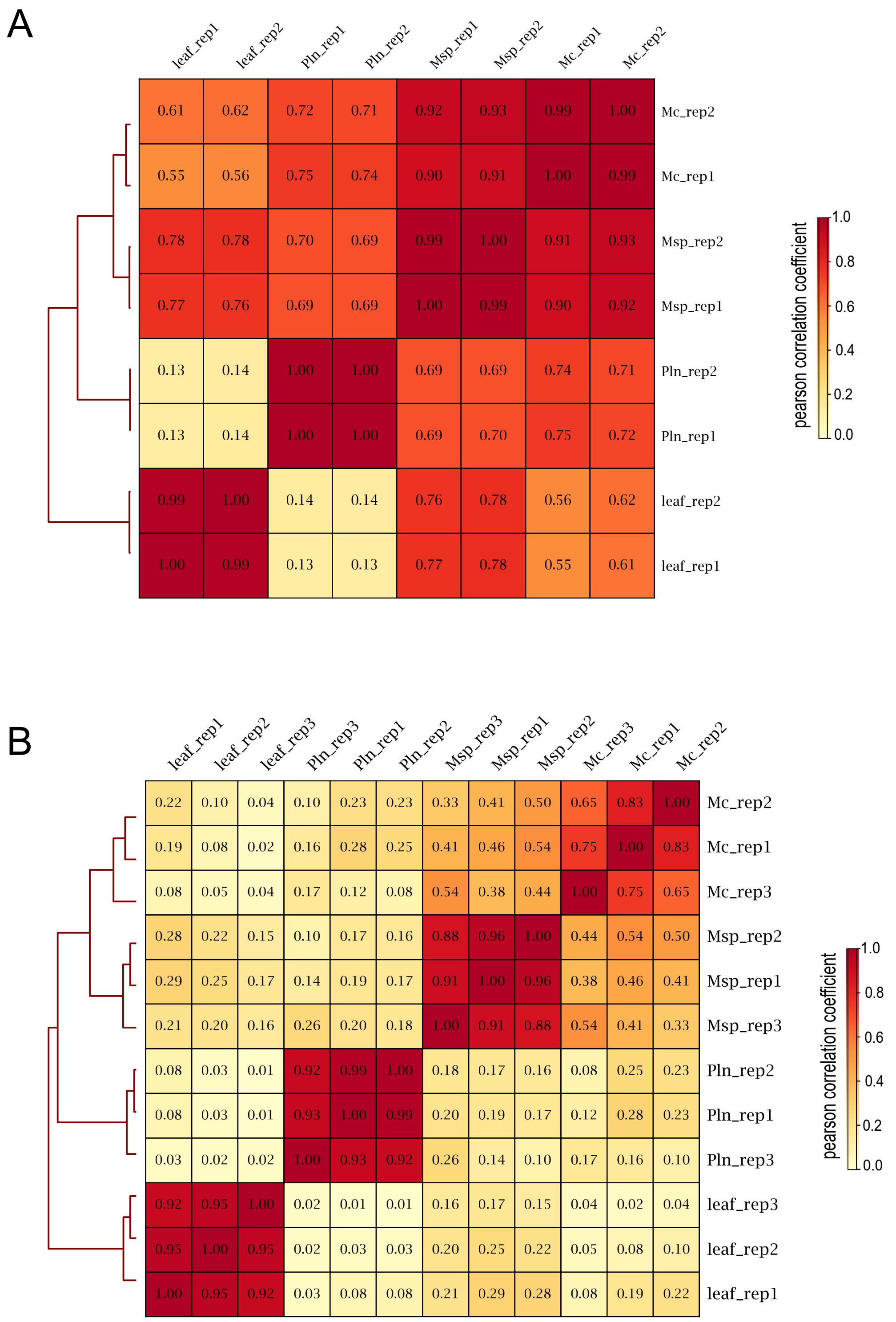

Figure S1. The sample correlations for WGBS and RNA-seq analysis.
A, Heatmap showing the sample correlation for WGBS analysis. B, Heatmap showing the sample correlation for RNA-seq analysis.
Mc, meiocyte; Msp, microspore; Pln, pollen.

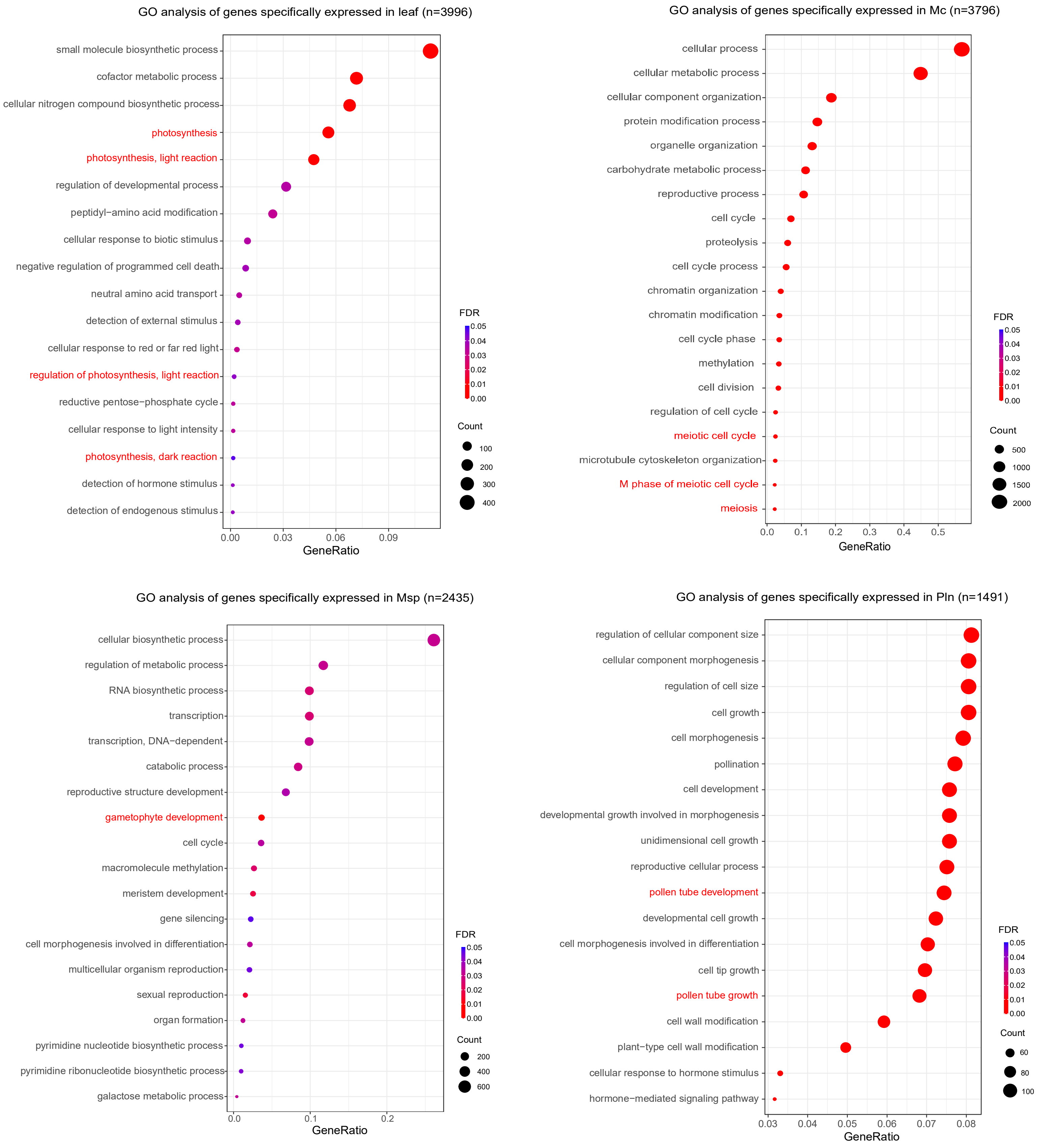

Figure S2. Specifically expressed genes enriched in different cell types.
Bubble Diagrams showing the Gene Ontolog analysis of genes specifically expressed in leaf, meiocyte, microspore and pollen. Biological processes specific to each cell type were highlighted in red. From the differentially expressed genes, the genes that were specifically expressed in each cell type were selected and then analyzed by GO, which showed the processes of photosynthesis, meiosis, gametophyte development, and pollen tube development were specifically present in leaf, meiocyte, microspore and pollen, respectively, thus determining the specificity of each cell type. Three biologic replicates were performed for RNA-seq with sex cells or leaf tissue (see Methods). n, the number of genes specifically expressed in each cell type. Mc, meiocyte; Msp, microspore; Pln, pollen.

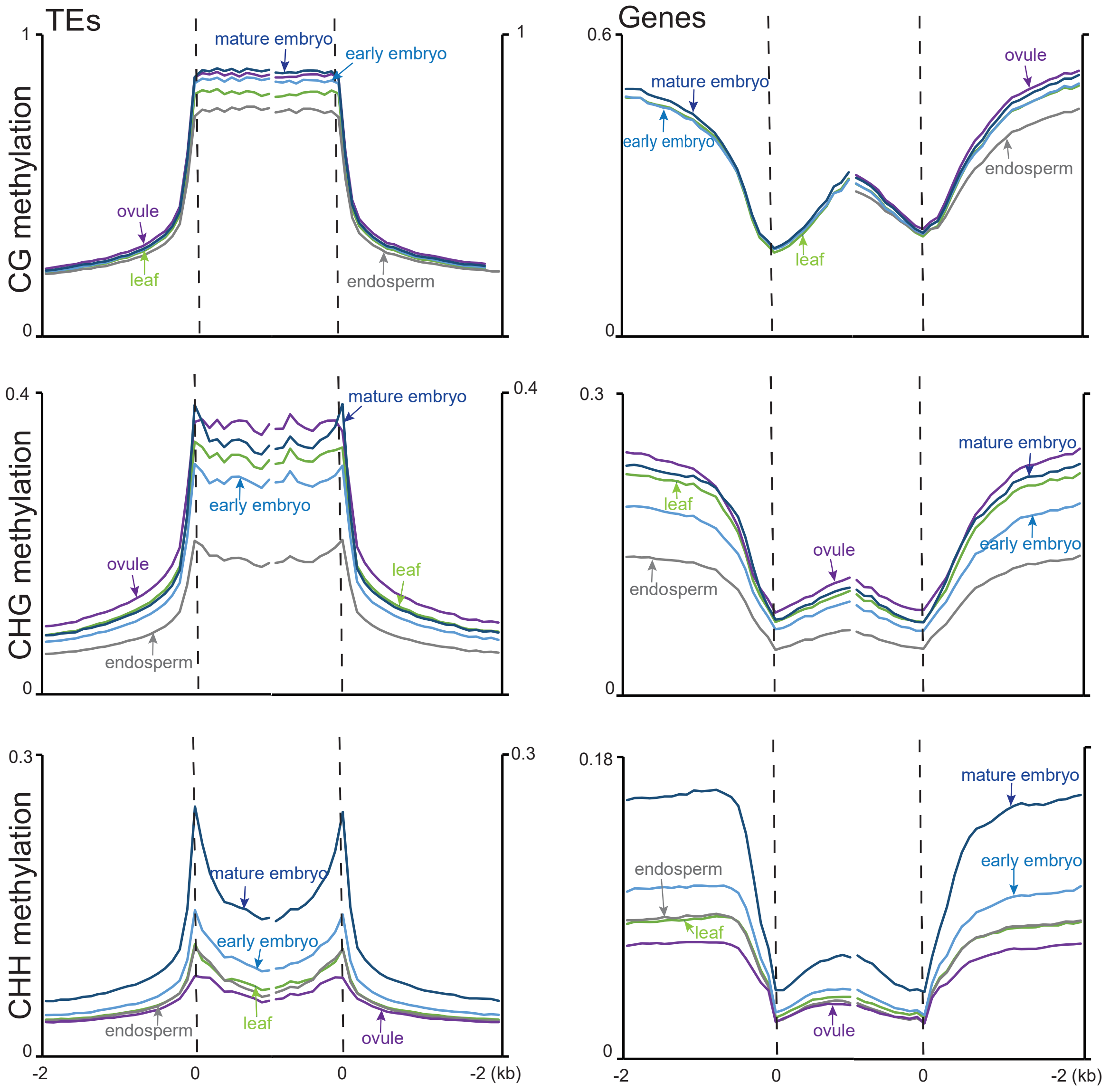

Figure S3. DNA methylation of transposons and genes in R-o-18.
R-o-18 transposons or genes were aligned at the 5′ and 3′ ends (dashed lines) with the average methylation in the CG, CHG or CHH context for each 100-bp interval are plotted for ovule, early embryo, mature embryo, endosperm and leaf. Three biologic replicates were analyzed for WGBS with ovule, endosperm or leaf tissue, and two biologic replicates were analyzed for WGBS with early or mature embryo in R-o-18[25].

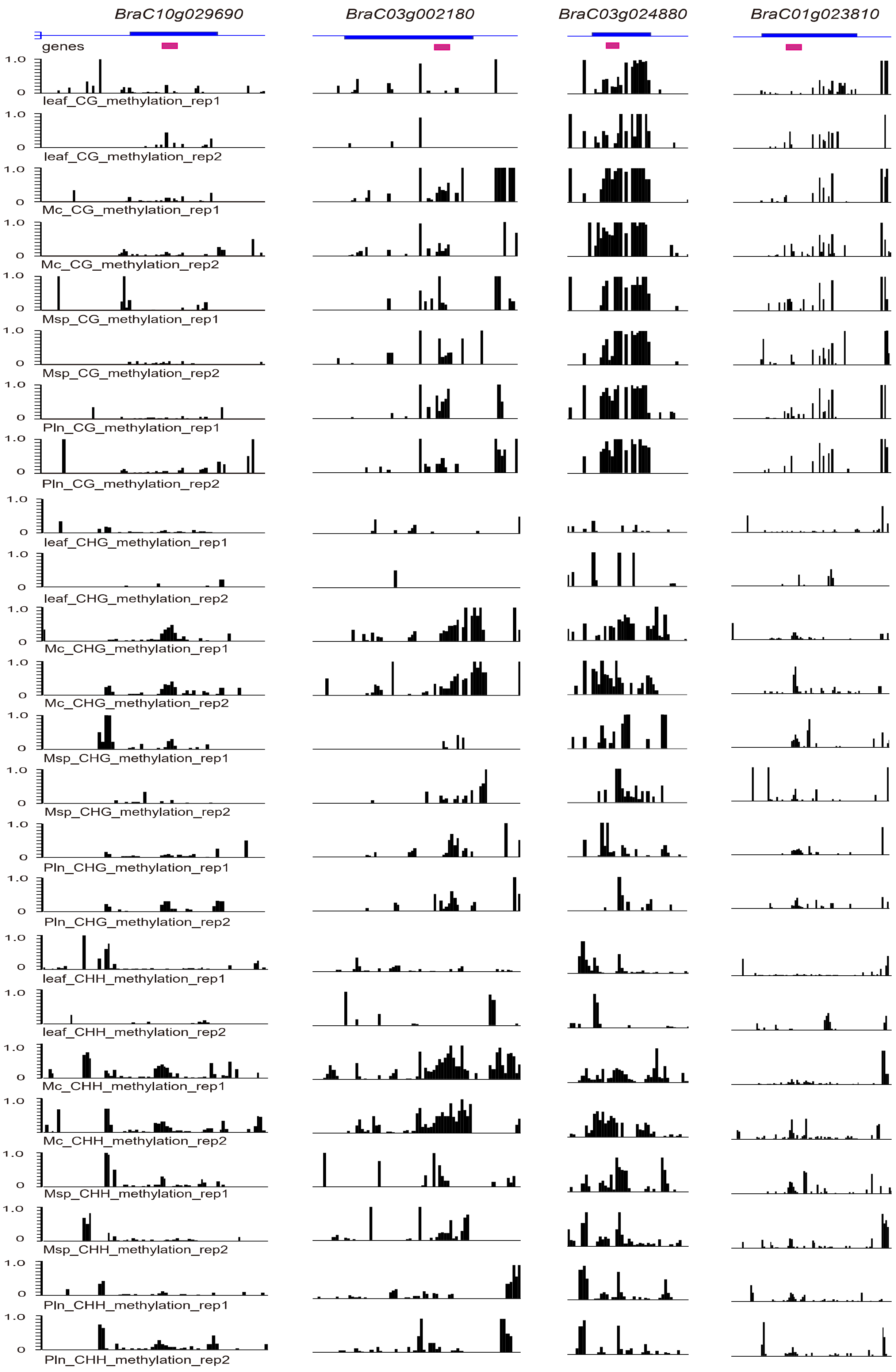

Figure S4. The CG/CHG/CHH methylation pattern of SLM-like loci targeted genes in male germline.
Snapshots of CG/CHG/CHH methylation at *BraC10g029690*, *BraC03g002180*, *BraC03g024880*, *BraC01g023810* genes. SLM-like loci were highlighted in magenta. Two biologic replicates of WGBS were showed. Mc, meiocyte; Msp, microspore; Pln, pollen.

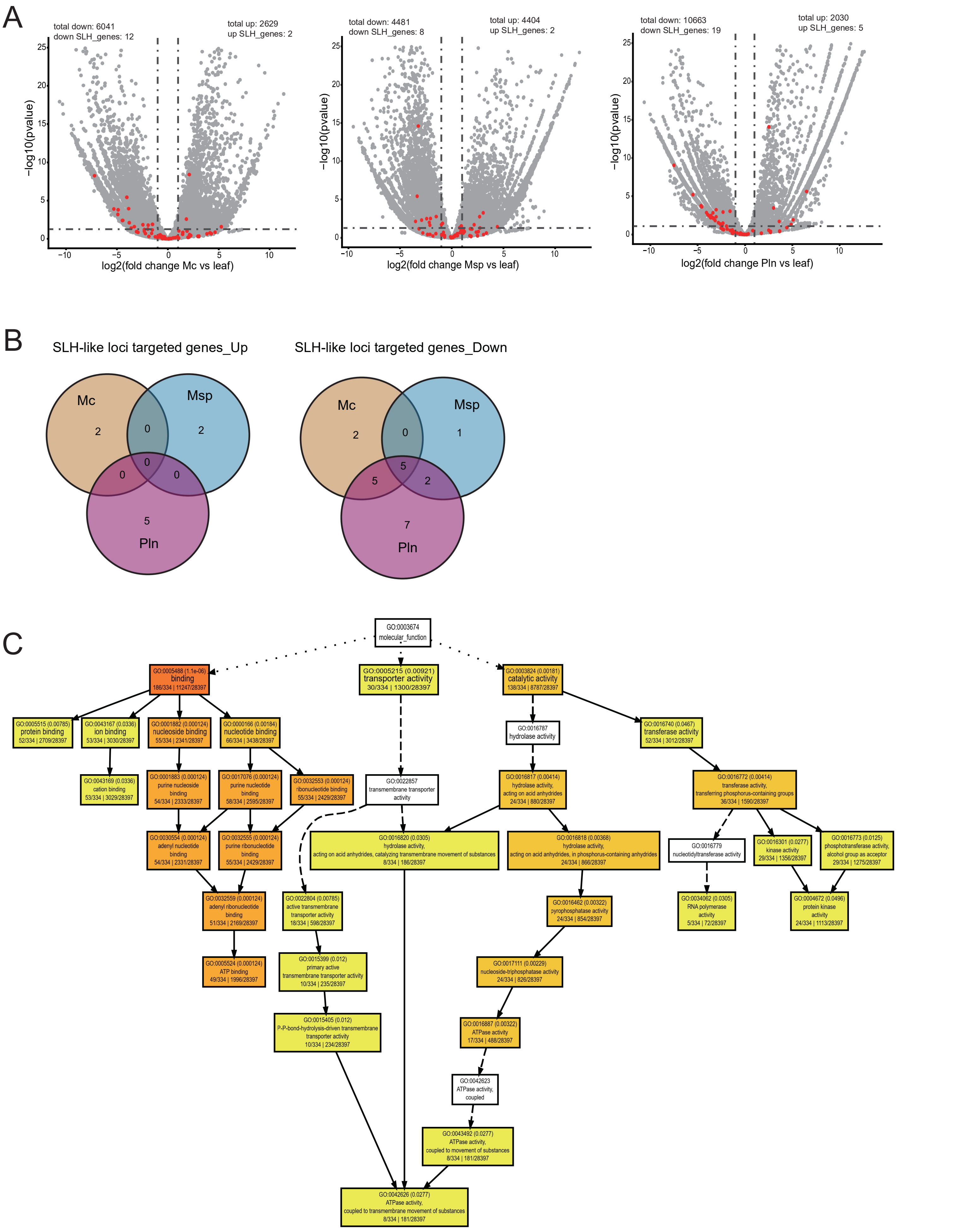

Figure S5. SLM/SLH-like loci target and may affect genes expression in male germline.
A, Volcano plot showing the differentially expressed genes (DEGs) in meiocyte, microspore and pollen compared with leaf with the SLH-like loci targeted genes highlighted in red. The vertical and horizontal dashed lines show the cutoff of the fold change > 2 and p-vaule < 0.05, respectively; total down/up, the number of total significantly down/up -regulated genes in each group; down/up SLH_genes, the number of significantly down/up-regulated SLH-like loci targeted genes in each group. B, Veen diagrams showing the overlap of up-regulated and down-regulated genes targeted by SLH-like loci in meiocyte, microspore and pollen. Three biologic replicates were performed for RNA -seq with sex cells or leaf tissue (see Methods). Mc, meiocyte; Msp, microspore; Pln, pollen. C, Graph showing the Gene Ontology analysis of SLM-like loci targeted genes.

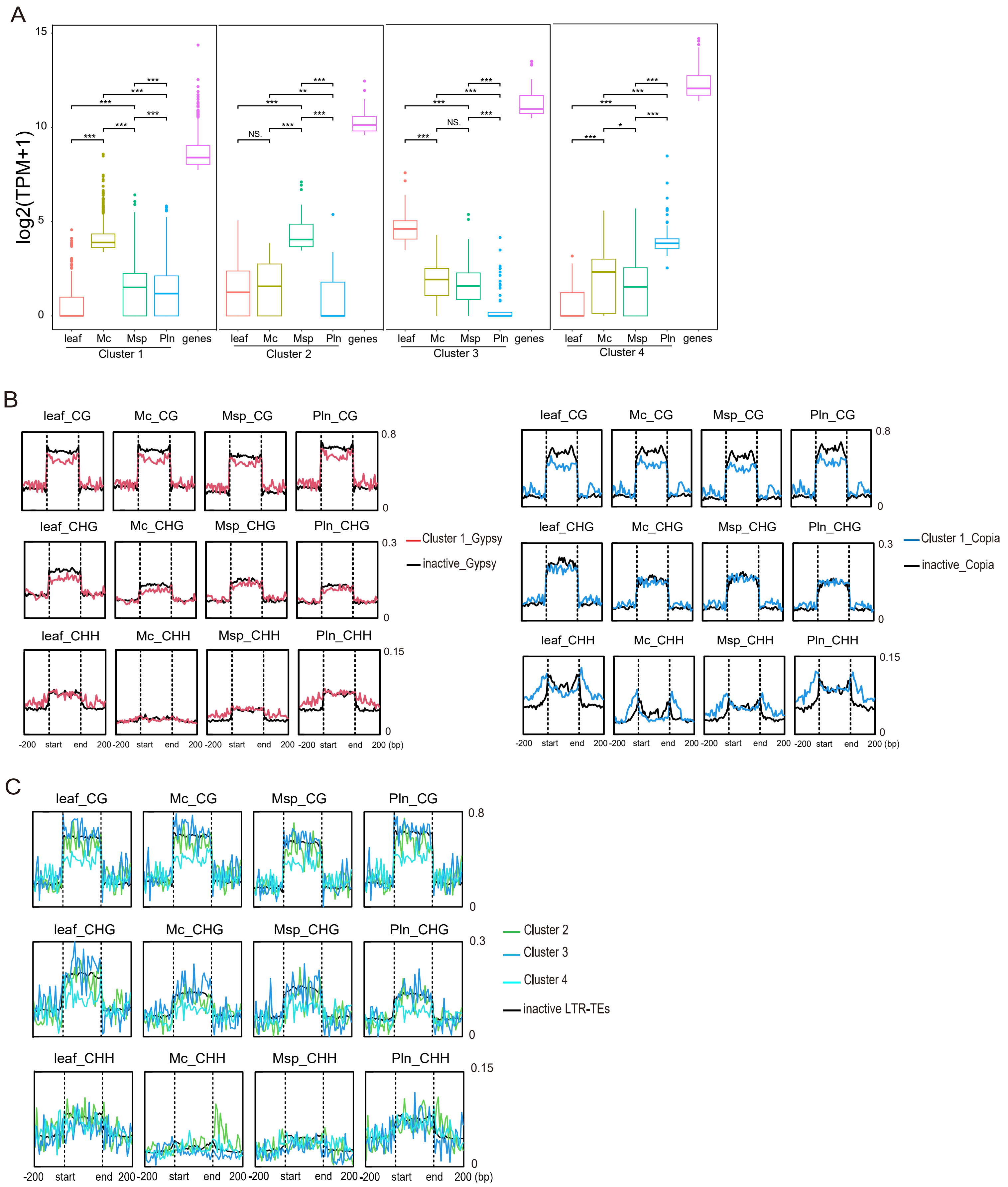

Figure S6. The surrounding DNA methylation of active and inactive LTR-TEs in K2 male germline.
A, Boxplot showing the expression levels of Cluster 1, Cluster 2, Cluster 3 and Cluster 4 LTR-TEs in leaf, meiocyte, microspore and pollen of K2, and the expression levels of genes highly expressed in leaf, meiocyte, microspore and pollen, respectively. *P < 0.1; **P <0.01; ***P < 0.001; NS., no significance. B, Curved line graph displaying the average methylation ratio in three contexts around the Cluster1_Gypsy and inactive_Gypsy (left panel), Cluster1_Copia and inactive_Copia (right panel) in leaf, meiocyte, microspore and pollen. Two biologic replicates were performed for WGBS with sex cells or leaf tissue in K2 (see Methods). C, Curved line graph displaying the average CG/CHG/CHH methylation ratio around the Cluster 2, 3, 4 and inactive LTR-TEs in leaf, meiocyte, microspore and pollen. Mc, meiocyte; Msp, microspore; Pln, pollen.

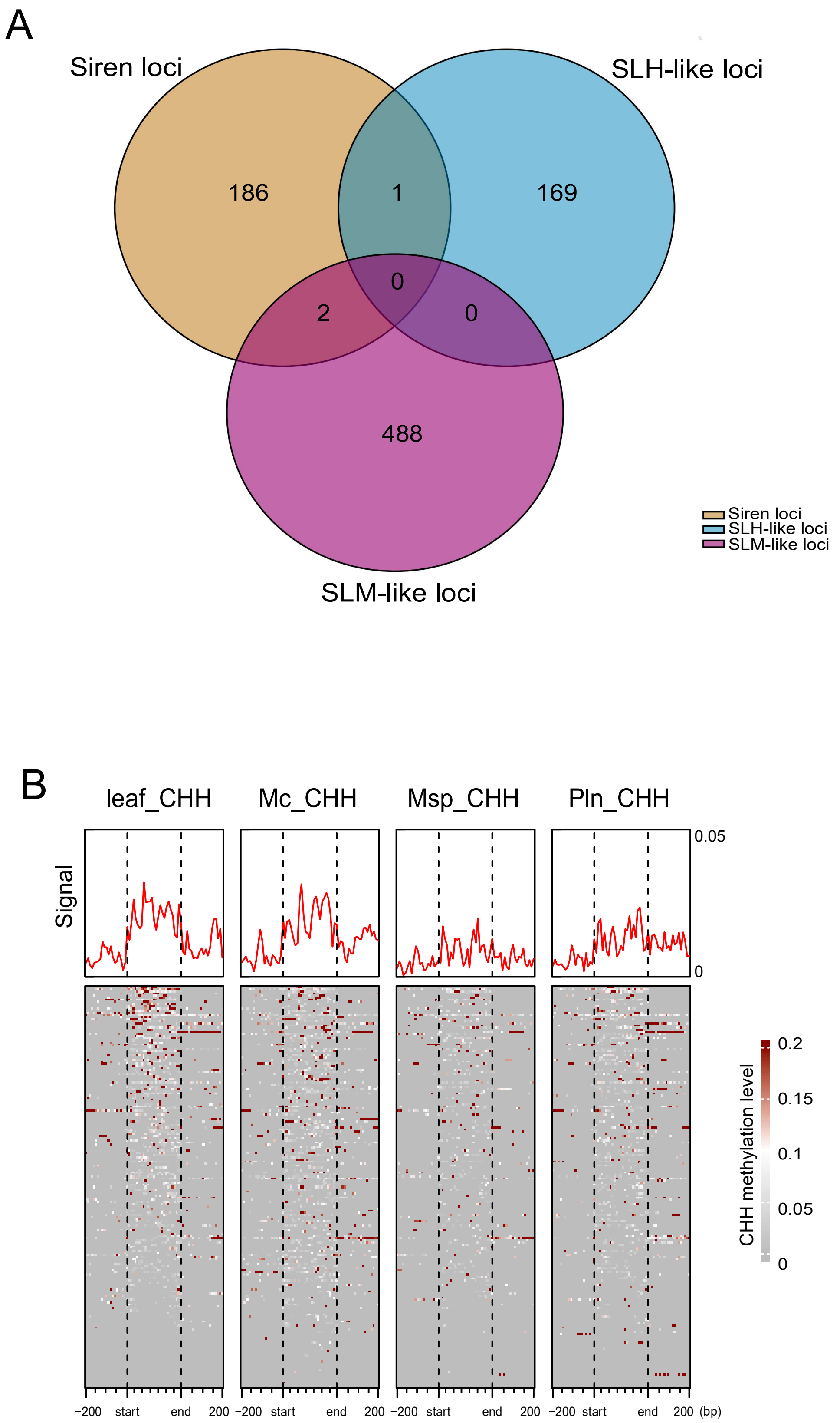

Figure S7. CHH methylation in the homologous sites of Siren loci in K2.
A, Venn diagram showing the overlapping result of the homologous sites of Siren loci, SLM-like loci and SLH-like loci in K2. B, Methylation profile for the homologous sites of Siren loci in leaf, meiocyte, microspore and pollen of K2. Curved line graph displaying the average CHH methylation ratio in the homologous sites of Siren loci in leaf, meiocyte, microspore and pollen of K2 (top). Heatmap showing CHH methylation level in the homologous sites of Siren loci in leaf, meiocyte, microspore and pollen of K2 (bottom). Two biologic replicates were performed for WGBS with sex cells or leaf tissue in K2 (see Methods). Mc, meiocyte; Msp, microspore; Pln, pollen.
